# Structure-based and ligand-based virtual screening of novel methyltransferase inhibitors of the dengue virus

**DOI:** 10.1186/1471-2105-12-S13-S24

**Published:** 2011-11-30

**Authors:** See Ven Lim, Mohd Basyaruddin A Rahman, Bimo A Tejo

**Affiliations:** 1 Department of Chemistry, Faculty of Science, Universiti Putra Malaysia, 43400 UPM Serdang, Malaysia; 2 Structural Biology Research Centre, Malaysia Genome Institute, UKM-MTDC Technology Centre, Universiti Kebangsaan Malaysia, 43600 UKM Bangi, Malaysia

## Abstract

**Background:**

The dengue virus is the most significant arthropod-borne human pathogen, and an increasing number of cases have been reported over the last few decades. Currently neither vaccines nor drugs against the dengue virus are available. NS5 methyltransferase (MTase), which is located on the surface of the dengue virus and assists in viral attachment to the host cell, is a promising antiviral target. In order to search for novel inhibitors of NS5 MTase, we performed a computer-aided virtual screening of more than 5 million commercially available chemical compounds using two approaches: i) structure-based screening using the crystal structure of NS5 MTase and ii) ligand-based screening using active ligands of NS5 MTase. Structure-based screening was performed using the LIDAEUS (LIgand Discovery At Edinburgh UniverSity) program. The ligand-based screening was carried out using the EDULISS (EDinburgh University LIgand Selection System) program.

**Results:**

The selection of potential inhibitors of dengue NS5 MTase was based on two criteria: the compounds must bind to NS5 MTase with a higher affinity than that of active NS5 MTase ligands, such as ribavirin triphosphate (RTP) and *S*-adenosyl-L-homocysteine (SAH); and the compounds must interact with residues that are catalytically important for the function of NS5 MTase. We found several compounds that bind strongly to the RNA cap site and the *S*-adenosyl-L-methionine (SAM) binding site of NS5 MTase with better binding affinities than that of RTP and SAH. We analyzed the mode of binding for each compound to its binding site, and our results suggest that all compounds bind to their respective binding sites by interacting with, and thus blocking, residues that are vital for maintaining the catalytic activity of NS5 MTase.

**Conclusions:**

We discovered several potential compounds that are active against dengue virus NS5 MTase through virtual screening using structure-based and ligand-based methods. These compounds were predicted to bind into the SAM binding site and the RNA cap site with higher affinities than SAH and RTP. These compounds are commercially available and can be purchased for further biological activity tests.

## Background

Dengue fever is an ancient disease that was first recorded in a Chinese encyclopedia of diseases and symptoms published during the Chin Dynasty (265 to 420 AD) [1]. Referred to as “water poison” in ancient Chinese medical literatures due to its association with flying insects bred in clean water, modern science has now confirmed that dengue fever is a viral disease transmitted between human hosts by *Aedes* mosquitoes, particularly *Aedes aegypti*. The ability of *Aedes aegypti* to adapt well to urban living environments plays a significant role in the outbreak of dengue fever [2]. Currently, dengue fever is the most important tropical infectious disease after malaria, and more than 100 countries have reported infections, especially countries in tropical and subtropical regions [3]. An estimated 100 million cases of dengue fever occur annually. Of these, 500,000 cases require hospitalization, and 25,000 are fatal [1,4], particularly in developing and underdeveloped countries where access to healthcare facilities is limited.

**Figure 1:**
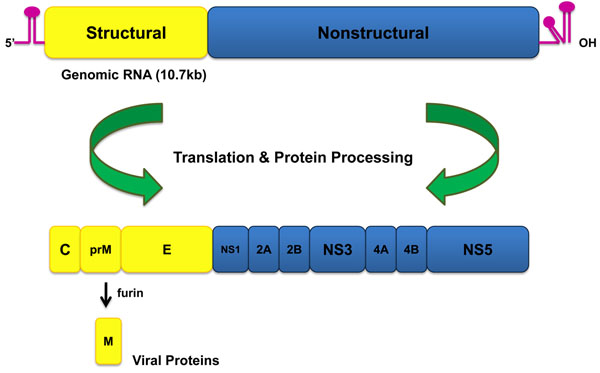
Schematic figure of *Flavivirus* RNA and its translation into proteins involved in the DV lifecycle.

In general, the dengue virus (DV) is a plus-strand RNA virus of the *Flavivirus* genus of the *Flaviviridae* family [5]. The DV has an approximately 50 nm envelope and contains a 10.7 kb single strand RNA that is translated into a single polyprotein followed by co-translational cleavage into 10 mature proteins. These 10 mature proteins consist of three structural proteins (capsid (c), premembrane (prM), envelope (E)) and seven nonstructural proteins (NS1, NS2A, NS2B, NS3, NS4A, NS4B, and NS5) (Figure 1) [6]. The nonstructural proteins are involved in evading innate immune responses, virion assembly, and the replication of the genome. The structural proteins play a role in the formation of the viral particle [6-8]. To date, the enzymatic activities of NS3 and NS5 are the best characterized among the non-structural DV proteins. Based on a number of studies, the methyltransferase (MTase) domain of the DV non-structural protein NS5 (NS5 MTase) is thought to be a promising antiviral target [9-12].

The stability of dengue viral mRNA and the efficient translation are closely related to its cap structure. The 5′ end of the dengue RNA has a type 1 cap structure (me^7^-GpppA-me^2^), whose formation requires NS3 and NS5 enzymatic reactions [13]. NS5 MTase methylates the guanosine cap at the N7 position by transferring a methyl group from *S*-adenosyl-L-methionine (SAM) followed by methylation of the 2′-OH position of the first ribose nucleotide [14]. A side product, *S*-adenosyl-L-homocysteine (SAH), is produced from N7 and 2′-O methylations [15]. Although MTase exhibits two distinct methylations, it has only single SAM binding site in its crystal structure [16]. It has been suggested that the substrate GpppA-RNA must be re-positioned to accept the N7 and 2′-O methyl groups from SAM during the two methylation reactions. There are two binding sites connected by a Y-shaped cleft. The first site is the SAM (methyl donor) binding site, and the second site is a shallow pocket of the RNA cap site [17].

**Figure 2:**
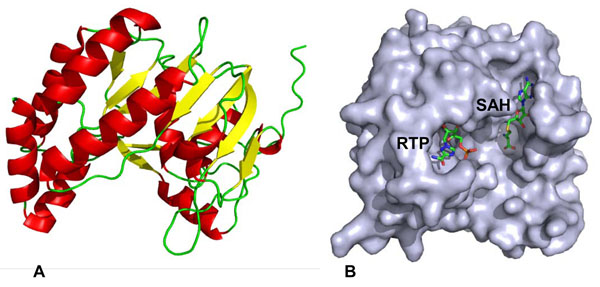
**Structure of dengue NS5 MTase [PDB:1R6A].** (a) Ribbon representation of NS5 MTase; α-helix is illustrated in red and β-sheet is illustrated in yellow. (b) Surface representation of NS5 MTase with RTP and SAH inside the RNA cap site and SAM binding site, respectively.

NS5 MTase has a globular shape with a seven-stranded central β-sheet surrounded by six helices (Figure 2a). The crystal structure of NS5 MTase with SAH and a nucleoside analogue, ribavirin triphosphate (RTP), is shown in Figure 2b. Based on the crystal structure, there are two complexes assigned to two binding sites. One site is for SAH and the methyl donor, SAM, and the second site is situated on an N-terminal subdomain for guanosine-5′-triphosphate (GTP) analogues [18].

Currently, there are no vaccines or drugs to inhibit DV replication and transmission. The presence of four serotypes of the DV (DV1 – DV4) has hampered the efforts to develop effective drugs or vaccines against the DV [19]. Infection with one DV serotype will result in life-long immunity to that serotype; however, sequential infection by other serotypes of the DV will result more severe symptoms. Dengue hemorrhagic fever (DHF) and dengue shock syndrome (DSS) typically occur through antibody-mediated disease enhancement (ADE), either from previous DV infection [20] or from vaccine-induced ADE [21]. The unique properties of the DV make the design and discovery of effective vaccines and drugs against the DV extremely challenging.

In this work, we performed a computer-aided virtual screen of more than 5 million commercially available chemical compounds to find potential inhibitors of NS5 MTase. We utilized two different approaches: i) structure-based virtual screening using the crystal structure of NS5 MTAse [PDB:1R6A] and ii) ligand-based virtual screening using active ligands of NS5 MTase (ribavirin and SAH). Structure-based compound screening was performed using the LIDAEUS (LIgand Discovery At Edinburgh UniverSity) program [22], which maps the active site of the receptor and screens it against approximately 5 million commercially available chemical compounds in its database. Meanwhile, ligand-based compound screening was carried out using the EDULISS (EDinburgh University LIgand Selection System) program [23], which utilizes known natural substrates of NS5 MTase to find structurally similar compounds from its database. We discovered several compounds that fit into the two binding sites of NS5 MTase with a higher binding affinity than its active ligands. These compounds can be further tested to confirm their biological activities against the DV.

## Results

In this work, we employed two different approaches to virtually screen potential inhibitors against dengue virus NS5 MTase. A structure-based virtual screen was performed using LIDAEUS to generate a map based on the NS5 MTase binding site. This map was used to screen approximately 5 million compounds in the LIDAEUS database for those that fit into the binding site map. Meanwhile, a ligand-based virtual screen was done using the UFSRAT (Ultra Fast Shape Recognition with Atom Types) algorithm implemented in EDULISS to search for compounds that are structurally similar to the two active ligands of NS5 MTase, RTP and SAH.

We then selected the top 500 highest ranked compounds from the EDULISS and LIDAEUS screenings for further molecular docking experiments using AutoDock Vina. Even though LIDAEUS analyzed the initial docking of millions of compounds in its database to methyltransferase, it uses a course-grained docking tool that is essentially rigid body docking (rigid ligand and rigid protein). This is the only approach available for working with large datasets. LIDAEUS tries to get around the rigid-rigid limitation by representing multiple conformers of a small molecule within the dataset. Once LIDAEUS has identified the top small molecules, more accurate analysis/docking trials were performed using AutoDock Vina for flexible ligand docking. This is much more computationally expensive than the rigid-rigid docking carried out with LIDAEUS. Furthermore, EDULISS does not have the same automated docking functionality as LIDAEUS. AutoDock Vina was run to rank the proposed compounds from the EDULISS screen based on their binding affinities.

In total, we docked 2000 unique compounds from the LIDAEUS screen for molecules that potentially bind to SAM binding site (500 compounds) and RNA cap site (500 compounds) and from the EDULISS screen using RTP (500 compounds) and SAH (500 compounds) as templates. Binding affinity calculations for all 2000 compounds were carried out using an algorithm implemented in AutoDock Vina. We identified 40 compounds with the highest binding affinities to the SAM and RNA cap sites (Table 1) that have stronger binding to their respective binding sites than RTP and SAH. The rational basis of selecting compounds with binding affinities higher than that of RTP and SAH is that only compounds that bind to NS5 MTase with a higher affinity than its natural substrates can be developed as potential inhibitors. Only the compounds that are ranked first for the SAM and RNA cap sites will be discussed in detailed.

**Table 1:** Binding affinity and predicted binding pocket of docked compounds

Compound ID	Virtual screening	Binding pocket	Binding affinity (kcal/mol)
SPH1-103-799	EDULISS	RNA cap	-7.8
SPH1-101-102	EDULISS	RNA cap	-7.8
SPH1-027-074	EDULISS	RNA cap	-7.7
SPH1-014-180	EDULISS	RNA cap	-7.6
SPH1-047-692	EDULISS	RNA cap	-7.6
SPH1-000-259	EDULISS	RNA cap	-7.6
SPH1-013-274	EDULISS	RNA cap	-7.5
SPH1-013-272	EDULISS	RNA cap	-7.5
SPH1-013-273	EDULISS	RNA cap	-7.5
SPH1-013-271	EDULISS	RNA cap	-7.5
28SPH1-115-917	LIDAEUS	RNA cap	-7.9
35SPH1-021-288	LIDAEUS	RNA cap	-7.8
28SPH1-185-015	LIDAEUS	RNA cap	-7.8
28SPH1-149-718	LIDAEUS	RNA cap	-7.6
28SPH1-026-800	LIDAEUS	RNA cap	-7.6
28SPH1-024-902	LIDAEUS	RNA cap	-7.5
28SPH1-081-432	LIDAEUS	RNA cap	-7.5
29SPH1-063-033	LIDAEUS	RNA cap	-7.3
28SPH1-024-902	LIDAEUS	RNA cap	-7.3
28SPH1-348-781	LIDAEUS	RNA cap	-7.3
SPH1-007-088	EDULISS	SAM	-11.4
SPH1-111-460	EDULISS	SAM	-11.4
SPH1-177-492	EDULISS	SAM	-11.0
SPH1-020-782	EDULISS	SAM	-10.6
SPH1-016-182	EDULISS	SAM	-10.5
SPH1-159-983	EDULISS	SAM	-10.5
SPH1-129-393	EDULISS	SAM	-10.4
SPH1-031-492	EDULISS	SAM	-10.3
SPH1-107-521	EDULISS	SAM	-10.3
SPH1-297-578	EDULISS	SAM	-10.3
25SPH1-103-433	LIDAEUS	SAM	-10.0
25SPH1-103-428	LIDAEUS	SAM	-9.9
42SPH1-001-864	LIDAEUS	SAM	-9.8
25SPH1-103-428	LIDAEUS	SAM	-9.8
42SPH1-001-481	LIDAEUS	SAM	-9.5
42SPH1-001-925	LIDAEUS	SAM	-9.5
25SPH1-108-370	LIDAEUS	SAM	-9.3
42SPH1-013-393	LIDAEUS	SAM	-9.2
25SPH1-102-225	LIDAEUS	SAM	-9.1
25SPH1-102-901	LIDAEUS	SAM	-8.2

### Validation of the docking

**Figure 3:**
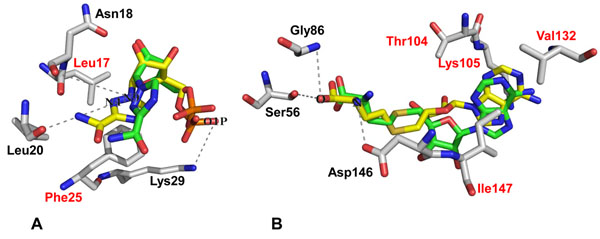
**Redocking of RTP and SAH into RNA cap site and SAM binding site.** (a) Crystallized conformation of RTP is shown in yellow carbons. The best redocked pose of RTP is shown in green carbons. (b) Crystallized conformation of SAH is shown in yellow carbons. The best redocked conformation of SAH is shown in green carbons. Residues with hydrophobic contacts with RTP and SAH are labeled in red and hydrogen bonds are drawn as dashed lines

In molecular docking, the size and center of the coordinates of the grid box need to be validated in order to ensure that ligands bind to the binding pocket in the correct conformation. In this work, docking validation was performed by redocking co-crystallized RTP and SAH into their respective binding sites. We found that the binding conformations of redocked RTP and SAH reproduced the binding modes of the co-crystallized ligands with binding affinities of -5.6 kcal/mol and -7.3 kcal/mol for RTP and SAH, respectively (Figure 3).

The RNA cap site of NS5 MTase is an open and shallow pocket. There are three polar interactions formed between RTP and the RNA cap site (Figure 3a). The backbone oxygen of Leu20 forms a hydrogen bond with the N4 nitrogen of RTP, and the backbone oxygen of Asn18 forms a hydrogen bond with the N9 nitrogen. Both N4 and N9 are located on a pseudobase moiety of RTP, which mimics the interaction between the guanine moiety of GDPMP (a GTP analogue) and NS5 MTase [15]. The backbone nitrogen of Lys29 forms a hydrogen bond with the O1P oxygen of RTP that further stabilizes RTP binding. A hydrophobic interaction between the pseudobase moiety of RTP and Leu17 of the RNA cap site was observed. The aromatic ring of Phe25 forms an aromatic stacking interaction with the pseudobase ring of RTP (Figure 3a).

The SAM binding pocket is thought to be more closed than the RNA cap site. The backbone oxygen of Ser56 and the nitrogen of Gly86 interact with the SAH molecule by forming hydrogen bonds with the carboxylic atoms of the homocysteine moiety of SAH. The carbonyl atom of the Asp146 side chain also forms a hydrogen bond with the amino group of the homocysteine moiety of SAH. In addition, there were four hydrophobic interactions between the hydrophobic side chains of Thr104, Lys105, Val132 and Ile147 in the SAM binding pocket with the adenosine moiety of the SAH molecule (Figure 3b).

Dong and colleagues [24] performed a biochemical and genetic characterization of the dengue virus NS5 MTase and identified important interactions between SAH and the surrounding residues of the SAM binding site. Asp131 interacts with the amino group of the adenosine moiety of SAH, while a hydrogen bond exists between the side chain of His110 and 2′-OH of the ribose moiety. Four residues (His110, Lys105, Asp131 and Ile147) were found to play an important role in N7 and 2-O′ methylations. That report emphasized the importance of the SAM binding site for both methylations. There are slight differences between our result and the result of Dong et al. in terms of the amino acids that are involved in the interaction between SAH and the SAM binding site. We identified three hydrogen bonds formed between SAH and Ser56, Gly86 and Asp146, which are not identified in Dong’s work. However, our results confirm that Lys105 and Ile147 form hydrophobic interactions with SAH.

### Inhibitors bound to RNA cap site

We performed molecular docking on 500 compounds obtained from the ligand-based EDULISS screen. These 500 compounds were ranked based on their binding affinities. The top ten compounds with binding affinites for the RNA cap site that are more negative than that of RTP are listed in Table 1. The two compounds that showed the strongest affinites for the RNA cap site of NS5 MTase were SPH1-103-799 and SPH1-101-102, each with a binding affinity of -7.8 kcal/mol. Meanwhile, 500 compounds from the structure-based LIDAEUS screen were also docked into the RNA cap site. The top 10 compounds with more negative binding affinities than that of RTP, indicating stronger binding, are listed in Table 1. The compound with the highest affinity discovered through the LIDAEUS screen was 28SPH1-115-917, with a binding affinity of -7.9 kcal/mol.

**Figure 4:**
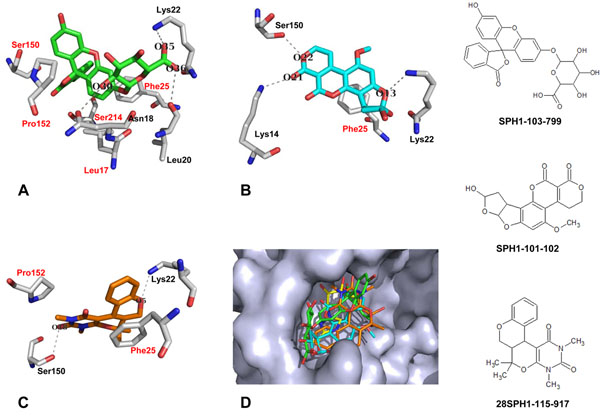
**Binding conformations of three compounds inside the RNA cap site.** (a) Compound SPH1-103-799 is shown in green carbons. (b) Compound SPH1-101-102 is shown in turquoise carbons. (c) Compound 28SPH1-115-917 is shown in orange carbons. Residues with hydrophobic contacts with the ligands are labeled in red. Hydrogen bonds are drawn as dashed lines. (d) Superimposition of three discovered compounds and RTP. Crystallized conformation of RTP is shown in yellow carbons, compound SPH1-103-799 is shown in green carbons, compound SPH1-101-102 is shown in turquoise carbons, and compound 28SPH1-115-917 is shown in orange carbons

Compound SPH1-103-799 (Figure 4a) forms three hydrogen bonds between oxygen O35 of the carbonyl group and the backbone nitrogen of Lys22, oxygen O36 of the hydroxyl group and the backbone oxygen of Leu20, and oxygen O30 of the hydroxyl group and the backbone oxygen of Asn18. Hydrophobic interactions occur between the aromatic rings of SPH1-103-799 and Leu17, Phe25, Ser150, Pro152, and Ser214. The second potential inhibitor, compound SPH1-101-102 (Figure 4b), also forms three hydrogen bonds between the oxygen O21 of the carbonyl group with the backbone nitrogen of Lys14, oxygen O22 with the side chain oxygen of Ser150, and oxygen O13 with the side chain nitrogen of Lys22. In contrast with compound SPH1-103-799, only one residue is involved in hydrophobic interaction between SPH1-101-102 and the RNA cap site at Phe25. Meanwhile, compound 28SPH1-115-917 (Figure 4c) forms a hydrogen bond between oxygen O18 of the carbonyl group and the side chain oxygen of Ser150. Likewise, the side chain nitrogen atom of Lys22 creates a hydrogen bond with oxygen O5. Two hydrophobic interactions are formed between the aromatic rings of 28SPH1-115-917 and Phe25 and Pro152.

Benarroch et al. [18], who studied the binding of RTP with the RNA cap site of NS5 MTase, suggested a list of residues that are significantly important for ligand binding: Lys14, Leu17, Asn18, Leu20, Phe25, Lys29, Ser150, Ser151, Pro152, Glu408, and Gly409. Our results show that compound SPH1-103-799 binds to the RNA cap site by forming interactions with eight residues: Leu17, Asn18, Leu20, Lys22, Phe25, Ser150, Pro152, and Ser214. These residues, with the exception of Lys22 and Ser214, interact with RTP in the crystal structure of NS5 MTase. Compounds SPH1-101-102 and 28SPH1-115-917 have fewer interactions with the RNA cap site. Only four residues are involved in the binding of these two compounds. Even though compounds SPH1-101-102 and 28SPH1-115-917 have fewer interactions with the RNA cap site compared to RTP, these compounds show stronger binding to the RNA cap site than RTP. There are several possible explanations for this effect: i) compound SPH1-101-102 creates three hydrogen bonds with the RNA cap site at different parts of the ligand, thus distributing the strength of the binding all over the ligand molecule; ii) compound 28SPH1-115-917 forms an aromatic stacking interaction with Phe25 that mimics the interaction of the pseudobase moiety of RTP with Phe25, and the ligand binding is strengthened by two hydrogen bonds with Lys22 and Ser150.

### Inhibitors bound to SAM binding site

After 500 compounds obtained from the ligand-based EDULISS screen were docked into the SAM binding site, 10 compounds with higher binding affinities for NS5 MTase than that of SAH were tabulated (Table 1). The two compounds with the strongest affinity for the SAM binding site were SPH1-007-088 and SPH1-111-460, each of which had a binding affinity of -11.4 kcal/mol. Meanwhile, 500 compounds obtained from the structure-based LIDAEUS screen were also docked into the SAM binding site, and the 10 best compounds are listed in Table 1. The best binder was compound 25SPH1-103-433 (-10.0 kcal/mol).

**Figure 5:**
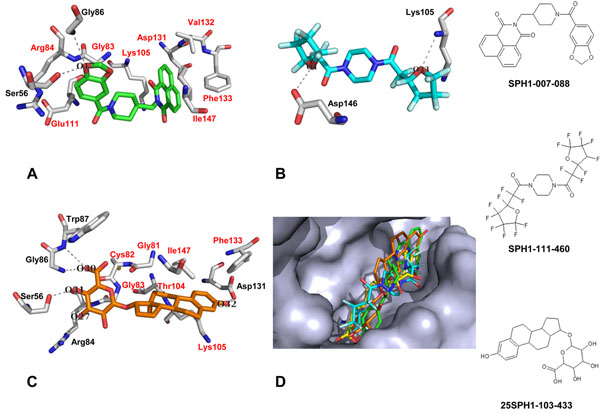
**Binding conformations of three compounds inside the SAM binding site.** (a) Compound SPH1-007-088 is shown in green carbons. (b) Compound SPH1-111-460 is shown in turquoise carbons. (c) Compound 25SPH1-103-433 is shown in orange carbons. Residues with hydrophobic contacts with the ligands are labeled in red. Hydrogen bonds are drawn as dashed lines. (d) Superimposition of three discovered compounds and SAH. Crystallized conformation of SAH is shown in yellow carbons, compound SPH1-007-088 is shown in green carbons, compound SPH1-111-460 is shown in turquoise carbons, and compound 25SPH1-103-433 is shown in orange carbons

Compound SPH1-007-088 (Figure 5a) forms two hydrogen bonds and several hydrophobic interactions with residues inside the SAM binding site. Two hydrogen bonds are formed between the side chain oxygen of Ser56 and the backbone nitrogen of Gly86 with oxygen O17. In addition, several hydrophobic interactions are formed between compound SPH1-007-088 with the hydrophobic side chains of Gly83, Arg84, Lys105, Glu111, Asp131, Val132, Phe133, and Ile147. As for compound SPH1-111-460 (Figure 5b), there are two hydrogen bonds formed with the SAM binding site. Oxygen O1 of SPH1-111-460 creates a hydrogen bond with the side chain oxygen of Asp146, whereas oxygen O33 forms a hydrogen bond with the backbone nitrogen of Lys105. Meanwhile, compound 25SPH1-103-433 (Figure 5c) forms five hydrogen bonds between oxygen O31 and the carbonyl oxygen atom of Ser56, oxygen O32 and the side chain oxygen atom of Asp131, oxygen O27 and the side chain nitrogen atom of Arg84, oxygen O30 and the backbone nitrogen atom of Trp87, as well as the backbone nitrogen atom of Gly86. In addition, several hydrophobic interactions were found between compound 25SPH1-103-433 and Gly81, Cys82, Gly83, Thr104, Lys105, Phe133, and Ile147.

Dong et al. [24] suggested that four residues are significantly important ligand binding in the SAM binding pocket: Lys105, His110, Asp131, and Ile147. Three of the compounds we discovered create one or more interactions with the four important residues in the SAM binding site. Compound SPH1-007-088 binds to Lys105, Asp131, and Ile147. Compound SPH1-111-460 binds to Lys105, and compound 25SPH1-103-433 interacts with Lys105, Asp131, and Ile147. Our results indicate that these three compounds can block the SAM binding site by binding to its critical residues.

## Discussion

Dengue and DHF remain pharmacologically neglected diseases [25] despite the high mortality rate, especially in developing countries where access to healthcare facilities is limited. Economic arguments have hampered the progress in discovering effective therapies for dengue [26]. The fact that dengue primarily affects poor communities prevents many pharmaceutical companies from making a serious effort to find the cure for this disease. Therefore, our research group has taken an initiative to be involved in the discovery of dengue chemotherapy by utilizing a limited laboratory setting similar to those commonly found in many developing countries.

We chose to perform a virtual screen of more than 5 million commercially available chemical compounds for two reasons. First, the cost of a screening campaign is sometimes prohibitive for laboratories in poor countries [27]. Therefore, virtual screening using computational methods may be performed to complement the “real” high-throughput screening (HTS) in less-funded laboratories. However, this approach has several disadvantages compared to HTS. For example, receptor flexibility is sometimes ignored in order to speed-up the computational screening. Second, the “drug universe” (including all the possible drug-like molecules) consists of 10^62^ molecules [28]. Thus, it is likely that potential molecules for dengue chemotherapy can be identified from the large universe of chemical compounds. To find potential chemotherapeutic compounds, the best strategy is not to synthesizing more compounds, but rather to use screening strategies to reduce the size of the molecular universe, as eloquently stated by Lahana in 1999: “when trying to find a needle in a haystack, the best strategy might not be to increase the size of the haystack” [28].

Performing virtual screening from a large database of chemical compounds requires access to multiprocessor computers, either in cluster or grid-based architectures. Research institutions in developed countries have launched a number of grid-based drug discovery efforts [29-31]; however, similar facilities often do not exist in developing countries. This fact motivated us to utilize two web-based drug-like compound databases (EDULISS and LIDAEUS) for the screening of commercially available chemical compounds as potential inhibitors of dengue NS5 MTase. The compounds screened from both databases were subsequently ranked based on their binding affinities to NS5 MTase using AutoDock Vina run on a single personal computer. The settings used in our experiments are suitable for most laboratories in developing countries with facilities that are inadequate for HTS and limited access to powerful multiprocessor computers.

In this work, we discovered several compounds that are potentially able to block the interaction between dengue NS5 MTase and its substrates, suggested that they may be able to stop the DV from replicating. The selection of potential compounds was based on their binding affinities. Compounds with stronger binding affinities for the RNA cap and SAM binding sites than that of SAH and RTP (NS5 MTase active ligands) were selected as potential inhibitors. In addition, the selected compounds must interact with residues that are catalytically important for the function of NS5 MTase. The mode of interaction between the compounds with the highest affinities and their binding sites was also discussed in detail.

### RNA cap site

It has been suggested that nucleoside analogues are among the best NS5 MTase inhibitors. These compounds are expected to bind to the RNA binding site based on the fact that the strongest interaction between guanosine-5′-triphosphate (GTP, the natural substrate for NS5 MTase) and the RNA cap site is formed by π-π stacking between Phe25 and the guanine moiety of GTP [14]. Ribavirin, a nucleoside analogue and a broad antiviral agent, has been shown to inhibit the replication of several DNA and RNA viruses [32], including the DV, although with weak activity (EC_50_ of 49 µg/mL) [33]. Ribavirin triphosphate (RTP) has also been shown to inhibit dengue NS5 MTase with low activity [34]. Figure 3a shows that RTP forms π-π stacking between Phe25 and the pseudobase moiety of RTP. Other nucleoside analogues have also been synthesized as antiviral agents, such as 5-ethynyl-1-*β*-D-ribofuranosylimidazole-4-carboxyamide [35], and 7-deaza-2′-*C*-methyladenosine [36].

Our screening results show that none of the discovered compounds can be classified as nucleoside analogues. Nevertheless, the three compounds (SPH1-103-799, SPH1-101-102, and 28SPH1-115-917) show better binding to the RNA cap site than RTP, as indicated by their binding affinities. Our results are in agreement with other findings showing that molecules without nucleoside moieties may have biological activity against NS5 MTase. Aurintricarboxylic acid was determined to be a potential inhibitor at the RNA cap site of NS5 MTase by binding tightly to Lys61 [37], a residue necessary for correct 2′-O methylation of the RNA [15]. Interestingly, this compound binds to the two binding sites on NS5 MTase (RNA cap site and SAM binding site) with different IC_50_ values (2.3 and 127 µM, respectively) [37]. Podvinec et al. also suggested two non-nucleoside analogues that act as potential inhibitors of NS5 MTase by binding to the RNA cap site with IC_50_ values of 4.9 and 7.1 µM [31].

### SAM binding site

In contrast to the RNA cap site, the SAM binding site is rather closed and long. This has hindered the development of several SAM analogues as NS5 MTase inhibitors, presumably due to off-target activity of the compounds [38,39]. Efforts have been made to overcome this problem. For example, Lim et al. synthesized SAH derivatives that bind selectively to the SAM binding site at submicromolar concentrations. These compounds are thought to specifically bind to dengue NS5 MTase and do not interfere with related human enzymes [40].

It has been suggested that the guanosine moiety is important for promoting the activity of inhibitors that bind to the SAM binding site due to the strong hydrophobic contacts between the guanosine ring and Lys105 and Ile147 [24]. Nevertheless, Podvinec et al. reported that two compounds with no guanosine moiety bound to the SAM binding site (IC_50_ of 4.4 and 9.5 µM) [31]. Our results confirm the findings by Podvinec et al. in that none of the compounds we discovered contain a guanosine moiety. All three compounds (SPH1-007-088, SPH1-111-460, and 25SPH1-103-433) bind to the SAM binding site by interacting with several residues important for MTase activity, such as Lys105, Asp131, and Ile147 [24].

Interestingly, the aromatic rings of compounds SPH1-007-088 and 25SPH1-103-433 create hydrophobic contacts with Phe133 (Figure 5a and 5c). Phe133 was not identified as one of the important residues for MTase catalytic activity by Dong et al [24]. However, in their recent publication, Lim et al. [40] identified a new hydrophobic cavity that consists of residues Phe133, Ile147, Gly148, Glu149, Arg160, Arg163, Val164, and Leu182. Phe133 blocks the SAM binding site in its native conformation, and this residue flips away from the cavity by 100 degrees upon binding to larger substrates. Compounds SPH1-007-088 and 25SPH1-103-433 bind to Phe133, suggesting that these compounds may restrain or prevent the hydrophobic cavity of the SAM binding site from opening.

## Conclusions

We have discovered several potential inhibitory compounds of dengue virus NS5 MTase through virtual screening using structure-based and ligand-based methods. These compounds were predicted to bind to the SAM and RNA cap sites with higher affinities than SAH and RTP. These compounds are commercially available and can be easily purchased for further biological activity tests.

All of the compounds we discovered in this work bind to their respective binding sites by creating hydrogen bonds and hydrophobic interactions with important residues in the binding pockets. We performed a detailed analysis of the atomic interactions between each potential compound and residues inside the SAM binding site and RNA cap site to identify which residues interact with the compounds. We have shown that the interaction between all compounds with the SAM binding site and RNA cap site are facilitated by hydrogen bonds and hydrophobic contacts with residues that are vital for NS5 MTase catalytic function. Therefore, these compounds may be used as leads for developing an effective dengue drug.

Our virtual screening experiments were performed in a laboratory setting that is commonly found in many developing countries. We decided not to develop an expensive multiprocessor computer architecture. Rather we utilized two free, web-based drug-like chemical compound databases (LIDAEUS and EDULISS) and the freely available docking program AutoDock Vina. The new algorithm implemented in AutoDock Vina permits the high-throughput docking of thousands of compounds in a shorter time compared to the classical AutoDock program. Thus, it only requires a single personal computer. Similar experiments can be carried out in laboratories with limited funding and facilities. Therefore, we hope that more researchers in developing countries can be actively involved in the global effort to fight dengue infection.

## Methods

### Software and program

PyMol (DeLano Scientific LLC, USA) and DS Visualizer (Accelrys, Inc., USA) were employed to visualize and modify the receptor and ligand structures. A molecular format conversion program, OpenBabelGUI, was used to prepare ligands for virtual screening and docking processes. The LIDAEUS (LIgand Discovery At Edinburgh UniverSity) [22] program was used to search for potential inhibitors based on a map of the NS5 MTase binding site by screening approximately 5 million commercially available chemical compounds. EDULISS (EDinburgh University LIgand Selection System) [23] is a chemical compound database that was used throughout this study to screen for potential inhibitors based on structural similarities with the known natural NS5 MTase substrates RTP and SAH. AutoDock Vina [41] was the primary docking program used in this work. The preparation of the MTase *pdbqt* file and determination of the grid box size were carried out using AutoDock Tools version 1.5.4 (The Scripps Research Institute, La Jolla, USA). The PaDel-ADV program (Department of Pharmacy, National University of Singapore, Singapore) was utilized as the interface to perform molecular docking of the chemical compounds obtained from LIDAEUS and EDULISS using AutoDock Vina. Post-docking analyses were carried out using the Program of Energetic Analysis of Receptor Ligand System (PEARLS) [42] and LigPlot [43].

### Preparation of NS5 MTase structure

The three-dimensional structure of NS5 MTase complexed with *S*-adenosyl-L-homocysteine (SAH) and ribavirin (RTP) was retrieved from the Protein Data Bank [PDB:1R6A]. The NS5 MTase structure was prepared for structure-based virtual screening and molecular docking processes by removing all sulfate ions and water molecules. Ligands (SAH and RTP) were removed and saved as two separate *pdb* files for further ligand-based virtual screening and control docking.

### Structure-based virtual screening

The web-based program LIDAEUS (http://opus.bch.ed.ac.uk/lidaeus/index.php) [22] was utilized to search for potential inhibitors against NS5 MTase by matching the binding site map of the receptor against chemical compounds in the database. First, the NS5 MTase structure was uploaded into LIDAEUS followed by SAH and RTP separately. LIDAEUS generated an energy map and site points on the MTase structure based on the positions where SAH and RTP reside in their binding pockets (the SAM binding site and RNA cap binding site, respectively). Then, approximately 5 million compounds were screened, and the top 500 compounds were saved for further molecular docking.

### Ligand-based virtual screening

Virtual screening based on the structure of NS5 MTase active ligands (SAH and RTP) was carried out using the web-based EDULISS program (http://eduliss.bch.ed.ac.uk/) [23]. Two-dimensional structures of SAH and RTP were used to search for similar compounds in the EDULISS database from the most common chemical suppliers (ChemBridge, MayBridge, PubChem, Sigma-Aldrich, Salor, Fluka and Specs), covering approximately one million chemical compounds. The concept of the similarity search employed in EDULISS is based on Ultra Fast Shape Recognition with Atom Types (UFSRAT) [44]. The top 500 compounds with the highest structural similarity to SAH and RTP were used for further molecular docking analysis.

### Molecular docking

Molecular docking was performed using AutoDock Vina [41]. Autodock Vina was used due to its accuracy and it speed, which is approximately two orders of magnitude faster than its predecessor, AutoDock 4 [45]. AutoDock Tools was utilized to prepare the input *pdbqt* file for NS5 MTase and to set the size and the center of the grid box. Kollman charges and polar hydrogen atoms were added to the NS5 MTase structure. Because NS5 MTase contains two active sites, the grid box cavity size and center were set separately for both sites. The RNA cap site was set at 15.180 × -51.931 × 17.429 in the dimensions of x, y and z using 1.000Å spacing, while the SAM binding site was set at 18.002 × -43.995 × -1.503 using 1.000Å spacing. AutoDock Vina requires the *pdbqt* input files of ligands to be prepared using AutoDock Tools. Due to the number of molecules to be docked (2000 compounds), the PaDel-ADV program was utilized to automate the docking process and act as an interface to perform the molecular docking of those compounds using AutoDock Vina. The predicted binding affinity (kcal/mol), which indicates how strongly a ligand binds to the receptor, is calculated based on the scoring function used in AutoDock Vina. A more negative binding affinity indicates stronger binding. The scoring function in AutoDock Vina is divided into two parts: i) a conformation-dependent part that can be seen as a sum of intramolecular and intermolecular contributions, including steric, hydrophobic, and hydrogen bonding interactions, and ii) a conformation-independent part that depends on the number of rotatable bonds between heavy atoms in the ligand. Each contribution (steric, hydrophobic, hydrogen bonding, and number of rotatable bonds) is given a different weight in the AutoDock Vina scoring function [41]. The validation of docking was carried out by redocking the NS5 MTase active ligands (SAH and RTP) into their binding sites. The Program of Energetic Analysis of Receptor Ligand System (PEARLS) and LigPlot programs were used to do post-docking analyses.

## List of abbreviations

DV: dengue virus; GTP: guanosine-5′-triphosphate; MTase: methyltransferase; NS5: nonstructural protein 5; RTP: ribavirin triphosphate; SAH: *S*-adenosyl-L-homocysteine; SAM: *S*-adenosyl-L-methionine.

## Competing interests

The authors declare that they have no competing interests.

## Authors’ contributions

SVL conducted the experiments and drafted the manuscript. MBAR participated in the setting of computer resources and experimental design. BAT conceived of the study, participated in its design and coordination and helped to draft the manuscript. All authors read and approved the final manuscript.

## References

[B1] Gubler DJ Dengue and dengue hemorrhagic fever Clin Microbiol Rev 1998 11 3 480 496 9665979 10.1128/cmr.11.3.480 PMC88892

[B2] Ligon BL Dengue fever and dengue hemorrhagic fever: a review of the history, transmission, treatment, and prevention Semin Pediatr Infect Dis 2005 16 1 60 65 10.1053/j.spid.2004.09.013 15685151

[B3] Gratz NG Emerging and resurging vector-borne diseases Annu Rev Entomol 1999 44 51 75 10.1146/annurev.ento.44.1.51 9990716

[B4] Halstead SB Dengue Lancet 2007 370 9599 1644 1652 10.1016/S0140-6736(07)61687-0 17993365

[B5] Mackenzie JS Gubler DJ Petersen LR Emerging flaviviruses: the spread and resurgence of Japanese encephalitis, West Nile and dengue viruses Nat Med 2004 10 12 Suppl S98 109 15577938 10.1038/nm1144

[B6] Perera R Kuhn RJ Structural proteomics of dengue virus Curr Opin Microbiol 2008 11 4 369 377 10.1016/j.mib.2008.06.004 18644250 PMC2581888

[B7] Guo JT Hayashi J Seeger C West Nile virus inhibits the signal transduction pathway of alpha interferon J Virol 2005 79 3 1343 1350 10.1128/JVI.79.3.1343-1350.2005 15650160 PMC544142

[B8] Padmanabhan R Mueller N Reichert E Yon C Teramoto T Kono Y Takhampunya R Ubol S Pattabiraman N Falgout B Multiple enzyme activities of flavivirus proteins Novartis Found Symp 2006 277 74 84 discussion 84-76, 251-253 17319155 10.1002/0470058005.ch6

[B9] Podvinec M Schwede T Peitsch MC Schwede T, Peitsch MC Docking for neglected diseases as community efforts Computational Structural Biology: Methods and Applications 2008 Singapore: World Scientific Publishing 683 704

[B10] Luzhkov VB Selisko B Nordqvist A Peyrane F Decroly E Alvarez K Karlen A Canard B Qvist J Virtual screening and bioassay study of novel inhibitors for dengue virus mRNA cap (nucleoside-2'O)-methyltransferase Bioorg Med Chem 2007 15 24 7795 7802 10.1016/j.bmc.2007.08.049 17888664

[B11] Cleaves GR Dubin DT Methylation status of intracellular dengue type 2 40 S RNA Virology 1979 96 1 159 165 10.1016/0042-6822(79)90181-8 111410

[B12] Ray D Shah A Tilgner M Guo Y Zhao Y Dong H Deas TS Zhou Y Li H Shi PY West Nile virus 5'-cap structure is formed by sequential guanine N-7 and ribose 2'-O methylations by nonstructural protein 5 J Virol 2006 80 17 8362 8370 10.1128/JVI.00814-06 16912287 PMC1563844

[B13] Egloff MP Benarroch D Selisko B Romette JL Canard B An RNA cap (nucleoside-2'-O-)-methyltransferase in the flavivirus RNA polymerase NS5: crystal structure and functional characterization Embo J 2002 21 11 2757 2768 10.1093/emboj/21.11.2757 12032088 PMC125380

[B14] Geiss BJ Thompson AA Andrews AJ Sons RL Gari HH Keenan SM Peersen OB Analysis of flavivirus NS5 methyltransferase cap binding J Mol Biol 2009 385 5 1643 1654 10.1016/j.jmb.2008.11.058 19101564 PMC2680092

[B15] Zhou Y Ray D Zhao Y Dong H Ren S Li Z Guo Y Bernard KA Shi PY Li H Structure and function of flavivirus NS5 methyltransferase J Virol 2007 81 8 3891 3903 10.1128/JVI.02704-06 17267492 PMC1866096

[B16] Courageot MP Frenkiel MP Dos Santos CD Deubel V Despres P Alpha-glucosidase inhibitors reduce dengue virus production by affecting the initial steps of virion morphogenesis in the endoplasmic reticulum J Virol 2000 74 1 564 572 10.1128/JVI.74.1.564-572.2000 10590151 PMC111573

[B17] Lim SP Wen D Yap TL Yan CK Lescar J Vasudevan SG A scintillation proximity assay for dengue virus NS5 2'-O-methyltransferase-kinetic and inhibition analyses Antiviral Res 2008 80 3 360 369 10.1016/j.antiviral.2008.08.005 18809436

[B18] Benarroch D Egloff MP Mulard L Guerreiro C Romette JL Canard B A structural basis for the inhibition of the NS5 dengue virus mRNA 2'-O-methyltransferase domain by ribavirin 5'-triphosphate J Biol Chem 2004 279 34 35638 35643 10.1074/jbc.M400460200 15152003

[B19] Morens DM Antibody-dependent enhancement of infection and the pathogenesis of viral disease Clin Infect Dis 1994 19 3 500 512 10.1093/clinids/19.3.500 7811870

[B20] Kummerer BM Rice CM Mutations in the yellow fever virus nonstructural protein NS2A selectively block production of infectious particles J Virol 2002 76 10 4773 4784 10.1128/JVI.76.10.4773-4784.2002 11967294 PMC136122

[B21] Stevens AJ Gahan ME Mahalingam S Keller PA The medicinal chemistry of dengue fever J Med Chem 2009 52 24 7911 7926 10.1021/jm900652e 19739651

[B22] Taylor P Blackburn E Sheng YG Harding S Hsin KY Kan D Shave S Walkinshaw MD Ligand discovery and virtual screening using the program LIDAEUS Br J Pharmacol 2008 153 Suppl 1 S55 67 18037921 10.1038/sj.bjp.0707532 PMC2268042

[B23] Hsin KY Morgan HP Shave SR Hinton AC Taylor P Walkinshaw MD EDULISS: a small-molecule database with data-mining and pharmacophore searching capabilities Nucleic Acids Res 2011 39 Database issue D1042 1048 21051336 10.1093/nar/gkq878 PMC3013767

[B24] Dong H Chang DC Xie X Toh YX Chung KY Zou G Lescar J Lim SP Shi PY Biochemical and genetic characterization of dengue virus methyltransferase Virology 2010 405 2 568 578 10.1016/j.virol.2010.06.039 20655081

[B25] Pang T Vaccines for the prevention of neglected diseases--dengue fever Curr Opin Biotechnol 2003 14 3 332 336 10.1016/S0958-1669(03)00061-2 12849789

[B26] Halstead SB Deen J The future of dengue vaccines Lancet 2002 360 9341 1243 1245 10.1016/S0140-6736(02)11276-1 12401270

[B27] Subramaniam S Mehrotra M Gupta D Virtual high throughput screening (vHTS)--a perspective Bioinformation 2008 3 1 14 17 19052660 10.6026/97320630003014 PMC2586130

[B28] Lahana R How many leads from HTS? Drug Discov Today 1999 4 10 447 448 10.1016/S1359-6446(99)01393-8 10481138

[B29] Chang MW Lindstrom W Olson AJ Belew RK Analysis of HIV wild-type and mutant structures via in silico docking against diverse ligand libraries J Chem Inf Model 2007 47 3 1258 1262 10.1021/ci700044s 17447753

[B30] Kasam V Zimmermann M Maass A Schwichtenberg H Wolf A Jacq N Breton V Hofmann-Apitius M Design of new plasmepsin inhibitors: a virtual high throughput screening approach on the EGEE grid J Chem Inf Model 2007 47 5 1818 1828 10.1021/ci600451t 17727268

[B31] Podvinec M Lim SP Schmidt T Scarsi M Wen D Sonntag LS Sanschagrin P Shenkin PS Schwede T Novel inhibitors of dengue virus methyltransferase: discovery by in vitro-driven virtual screening on a desktop computer grid J Med Chem 2010 53 4 1483 1495 10.1021/jm900776m 20108931

[B32] Sidwell RW Huffman JH Khare GP Allen LB Witkowski JT Robins RK Broad-spectrum antiviral activity of Virazole: 1-beta-D-ribofuranosyl-1,2,4-triazole-3-carboxamide Science 1972 177 50 705 706 10.1126/science.177.4050.705 4340949

[B33] Gabrielsen B Phelan MJ Barthel-Rosa L See C Huggins JW Kefauver DF Monath TP Ussery MA Chmurny GN Schubert EM Synthesis and antiviral evaluation of N-carboxamidine-substituted analogues of 1-beta-D-ribofuranosyl-1,2,4-triazole-3-carboxamidine hydrochloride J Med Chem 1992 35 17 3231 3238 10.1021/jm00095a020 1507208

[B34] Crance JM Scaramozzino N Jouan A Garin D Interferon, ribavirin, 6-azauridine and glycyrrhizin: antiviral compounds active against pathogenic flaviviruses Antiviral Res 2003 58 1 73 79 10.1016/S0166-3542(02)00185-7 12719009

[B35] Koff WC Pratt RD Elm JL Jr. Venkateshan CN Halstead SB Treatment of intracranial dengue virus infections in mice with a lipophilic derivative of ribavirin Antimicrob Agents Chemother 1983 24 1 134 136 6684897 10.1128/aac.24.1.134 PMC185118

[B36] Olsen DB Eldrup AB Bartholomew L Bhat B Bosserman MR Ceccacci A Colwell LF Fay JF Flores OA Getty KL A 7-deaza-adenosine analog is a potent and selective inhibitor of hepatitis C virus replication with excellent pharmacokinetic properties Antimicrob Agents Chemother 2004 48 10 3944 3953 10.1128/AAC.48.10.3944-3953.2004 15388457 PMC521892

[B37] Milani M Mastrangelo E Bollati M Selisko B Decroly E Bouvet M Canard B Bolognesi M Flaviviral methyltransferase/RNA interaction: structural basis for enzyme inhibition Antiviral Res 2009 83 1 28 34 10.1016/j.antiviral.2009.03.001 19501254 PMC7127253

[B38] Vedel M Lawrence F Robert-Gero M Lederer E The antifungal antibiotic sinefungin as a very active inhibitor of methyltransferases and of the transformation of chick embryo fibroblasts by Rous sarcoma virus Biochem Biophys Res Commun 1978 85 1 371 376 10.1016/S0006-291X(78)80052-7 217377

[B39] Yebra MJ Sanchez J Martin CG Hardisson C Barbes C The effect of sinefungin and synthetic analogues on RNA and DNA methyltransferases from Streptomyces J Antibiot (Tokyo) 1991 44 10 1141 1147 10.7164/antibiotics.44.1141 1720117

[B40] Lim SP Sonntag LS Noble C Nilar SH Ng RH Zou G Monaghan P Chung KY Dong H Liu B Small molecule inhibitors that selectively block dengue virus methyltransferase J Biol Chem 2011 286 8 6233 6240 10.1074/jbc.M110.179184 21147775 PMC3057852

[B41] Trott O Olson AJ AutoDock Vina: improving the speed and accuracy of docking with a new scoring function, efficient optimization, and multithreading J Comput Chem 2010 31 2 455 461 19499576 10.1002/jcc.21334 PMC3041641

[B42] Han LY Lin HH Li ZR Zheng CJ Cao ZW Xie B Chen YZ PEARLS: program for energetic analysis of receptor-ligand system J Chem Inf Model 2006 46 1 445 450 10.1021/ci0502146 16426079

[B43] Wallace AC Laskowski RA Thornton JM LIGPLOT: a program to generate schematic diagrams of protein-ligand interactions Protein Eng 1995 8 2 127 134 10.1093/protein/8.2.127 7630882

[B44] Shave SR The development of high performances structure and ligand based virtual screening techniques PhD Thesis 2009 Edinburgh: University of Edinburgh

[B45] Morris GM Huey R Olson AJ Using AutoDock for ligand-receptor docking Curr Protoc Bioinformatics 2008 Chapter 8 Unit 8 14 19085980 10.1002/0471250953.bi0814s24

